# The Role of Genomics in Tracking the Evolution of Influenza A Virus

**DOI:** 10.1371/journal.ppat.1000566

**Published:** 2009-10-26

**Authors:** Alice Carolyn McHardy, Ben Adams

**Affiliations:** 1 Computational Genomics and Epidemiology, Max Planck Institute for Informatics, Saarbruecken, Germany; 2 Department of Mathematical Sciences, University of Bath, United Kingdom; The Scripps Research Institute, United States of America

## Abstract

Influenza A virus causes annual epidemics and occasional pandemics of short-term respiratory infections associated with considerable morbidity and mortality. The pandemics occur when new human-transmissible viruses that have the major surface protein of influenza A viruses from other host species are introduced into the human population. Between such rare events, the evolution of influenza is shaped by antigenic drift: the accumulation of mutations that result in changes in exposed regions of the viral surface proteins. Antigenic drift makes the virus less susceptible to immediate neutralization by the immune system in individuals who have had a previous influenza infection or vaccination. A biannual reevaluation of the vaccine composition is essential to maintain its effectiveness due to this immune escape. The study of influenza genomes is key to this endeavor, increasing our understanding of antigenic drift and enhancing the accuracy of vaccine strain selection. Recent large-scale genome sequencing and antigenic typing has considerably improved our understanding of influenza evolution: epidemics around the globe are seeded from a reservoir in East-Southeast Asia with year-round prevalence of influenza viruses; antigenically similar strains predominate in epidemics worldwide for several years before being replaced by a new antigenic cluster of strains. Future in-depth studies of the influenza reservoir, along with large-scale data mining of genomic resources and the integration of epidemiological, genomic, and antigenic data, should enhance our understanding of antigenic drift and improve the detection and control of antigenically novel emerging strains.

Influenza is a single-stranded, negative-sense RNA virus that causes acute respiratory illness in humans. In temperate regions, winter influenza epidemics result in 250,000–500,000 deaths per year; in tropical regions, the burden is similar [1],[2]. Influenza viruses of three genera or types (A, B, and C) circulate in the human population. Influenza viruses of the types B and C evolve slowly and circulate at low levels. Type A evolves rapidly and can evade neutralization by antibodies in individuals who have been previously infected with, or vaccinated against, the virus. As a result it regularly causes large epidemics. Furthermore, distinct reservoirs of influenza A exist in other mammals and in birds. Four times in the last hundred years these reservoirs have provided genetic material for novel viruses that have caused global pandemics [3]–[8].

The genome of influenza A viruses comprises eight RNA segments of 0.9–2.3 kb that together span approximately 13.5 kb and encode 11 proteins [9]. Segment 4 encodes the major surface glycoprotein called hemagglutinin (H), which is responsible for attaching the virus to sialic acid residues on the host cell surface and fusing the virus membrane envelope with the host cell membrane, thus delivering the viral genome into the cell (Figure 1). Segment 6 encodes another surface glycoprotein called neuraminidase (N), which cleaves terminal sialic acid residues from glycoproteins and glycolipids on the host cell surface, thus releasing budding viral particles from an infected cell [10]. Influenza A viruses are further classified into distinct subtypes based on the genetic and antigenic characteristics of these two surface glycoproteins. Sixteen hemagglutinin (H1–16) and nine neuraminidase subtypes (N1–9) are known to exist, and they occur in various combinations in influenza viruses endemic in aquatic birds [10],[11]. Viruses with the subtype composition H1N1 and H3N2 have been circulating in the human population for several decades. Of these two subtypes, H3N2 evolves more rapidly, and has until recently caused the majority of infections [1],[12],[13]. In the spring of 2009, however, a new H1N1 virus originating from swine influenza A viruses, and only distantly related to the H1N1 already circulating, gained hold in the human population. The emergence of this virus has initiated the first influenza pandemic of the twenty-first century [7],[14],[15].

**Figure 1:**
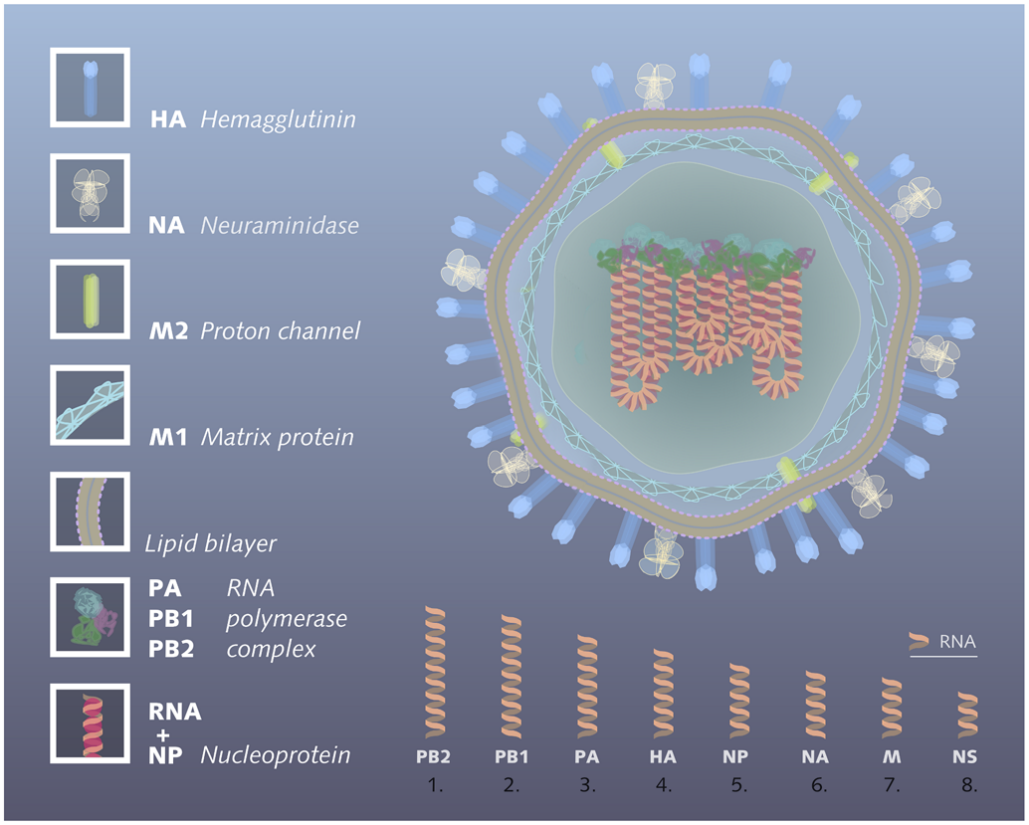
Schematic representation of an influenza A virion. Three proteins, hemagglutinin (HA, a trimer of three identical subunits), neuraminidase (NA, a tetramer of four identical subunits), and the M2 transmembrane proton channel (a tetramer of four identical subunits), are anchored in the viral membrane, which is composed of a lipid bilayer. The large, external domains of hemagglutinin and neuraminidase are the major targets for neutralizing antibodies of the host immune response. The M1 matrix protein is located below the membrane. The genome of the influenza A virus is composed of eight individual RNA segments (conventionally ordered by decreasing length, bottom row), which each encode one or two proteins. Inside the virion, the eight RNA segments are packaged in a complex with nucleoprotein (NP) and the viral polymerase complex, consisting of the PA, PB1, and PB2 proteins.

Hemagglutinin is about five times more abundant than neuraminidase in the viral membrane and is the major target of the host immune response [16]–[18]. Following exposure to the virus, whether by infection or vaccination, the host immune system acquires the capacity to produce neutralizing antibodies against the viral surface glycoproteins. These antibodies participate in clearing an infection and may protect an individual from future infections for many decades [19]. Five exposed regions on the surface of hemagglutinin, called epitope sites, are predominantly recognized by such antibodies [16],[17]. However, the human subtypes of influenza A continuously evolve and acquire genetic mutations that result in amino acid changes in the epitopes. These changes reduce the protective effect of antibodies raised against previously circulating viral variants. This “antigenic drift” necessitates frequent modification and readministration of the influenza vaccine to ensure efficient protection (Box 1).

**Box 1. Broadly Protective Vaccines**

Current influenza vaccines are based on detergent-inactivated viruses. They elicit antibodies with a narrow range of protection that target predominantly the variable regions of the hemagglutinin protein. Accordingly, the seasonal influenza vaccine includes one strain with segments of the surface proteins for each of the A/H1N1, A/H3N2 and B viruses, and it is updated every 1–3 years to match the predominant variants of influenza. Research into vaccines that offer broader protection across diverse subtypes and antigenic drift variants is ongoing [21], [59]–[61]. This research is particularly important with respect to the emergence of novel viruses with pandemic potential, such as the 2009 H1N1 virus. In such an event, the time period between the detection of the virus and the onset of a pandemic is too short to produce a specific vaccine for immediate vaccination of the population. Work in this area is focused on developing vaccines that elicit antibodies against conserved viral components, such as certain regions of hemagglutinin, neuraminidase, and the M2 proton channel in the viral membrane [60]. Other types of vaccines based on live attenuated viruses or plasmid DNA expression vectors, or supplemented with adjuvants, show promise in inducing a more broadly protective immune response [61].

To monitor for novel emerging strains, the World Health Organization (WHO) maintains a global surveillance program. A panel of experts meets twice a year to review antigenic, genetic, and epidemiological data and decides on the vaccine composition for the next winter season in the northern or southern hemisphere [20]. If an emerging antigenic variant is detected and judged likely to become predominant, an update of the vaccine strain is recommended. This “predict and produce” approach mostly results in efficient vaccines that substantially limit the morbidity and mortality of seasonal epidemics [21]. The recommendation has to be made almost a year before the season in which the vaccine is used, however, because of the time required to produce and distribute a new vaccine. Problems arise when an emerging variant is not identified early enough for an update of the vaccine composition [22]–[24]. Thus, gaining a detailed understanding of the evolution and epidemiology of the virus is of the utmost importance, as it may lead to earlier identification of novel emerging variants [20].

The development of high-throughput sequencing has recently provided large datasets of high-quality, complete genome sequences for viral isolates collected in a relatively unbiased manner, regardless of virulence or other unusual characteristics [9],[25]. Analyses of the genome sequence data combined with large-scale antigenic typing [26],[27] have given insights into the pattern of global spread, the genetic diversity during seasonal epidemics, and the dynamics of subtype evolution. Influenza data repositories such as the NCBI Influenza Virus Resource (http://www.ncbi.nlm.nih.gov/genomes/FLU/FLU.html) [28] and the Global Initiative on Sharing All Influenza Data (GISAID; http://platform.gisaid.org/) database [29] make the genomic information publicly available, together with epidemiological data for the sequenced isolates. The GISAID model for data sharing requires users to agree to collaborate with, and appropriately credit, all data contributors. A notable success of this initiative has been the contribution of countries, such as Indonesia and China, which have previously been reticent about placing data in the public domain. The WHO also supports the endeavor of rapid publication of all available sequences for influenza viruses and there is hope that comprehensive submission to public databases will soon become a reality [24],[30]. In the future, mining these resources and establishing a statistical framework based on epidemiological, antigenic, and genetic information could provide further insights into the rules that govern the emergence and establishment of antigenically novel variants and improve the potential for influenza prevention and control.

## Host Immune Evasion by Antigenic Drift and Shift

**Figure 2:**
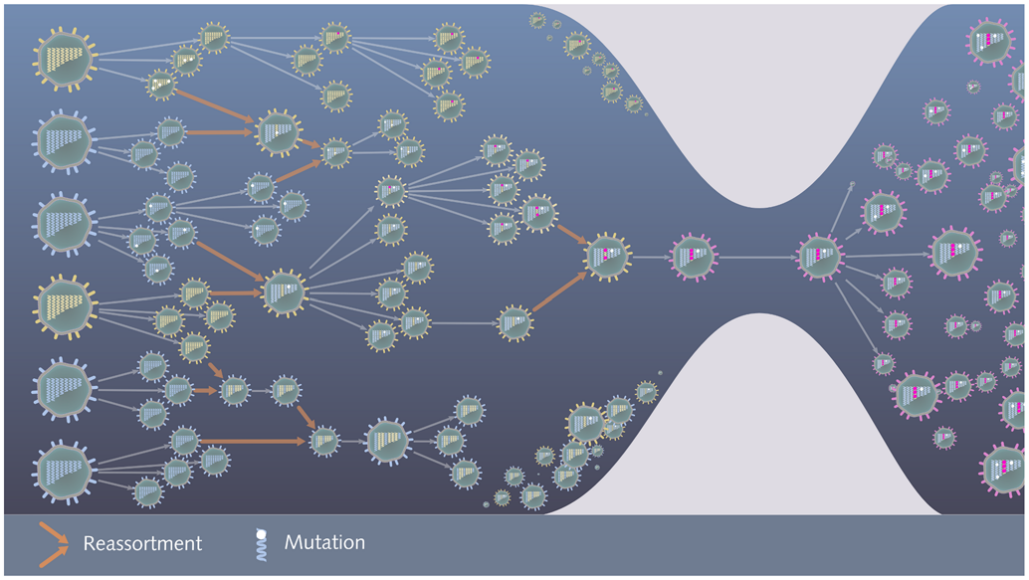
Generation of genetic diversity and antigenic drift in the evolution of human influenza A viruses. Blue and yellow viruses depict two antigenically similar strains of the same subtype circulating in the human population. The genetic diversity of the circulating viral population increases through mutation and reasssortment. Single white arrows indicate relationships between ancestral and descendant viruses. White marks on the segments indicate neutral mutations and red marks indicate mutations that affect the antigenic regions of the surface proteins. Incoming pairs of orange arrows indicate the generation of reassortants with segments from two different ancestral viruses. As these viruses continue to circulate, immunity against them builds up in the host population, represented here by the narrowing of the bottleneck. In parallel, viruses with mutations affecting the antigenic regions of the surface proteins accumulate in the viral population. At some point a novel antigenic drift variant, indicated by a red colored virus, which is less affected by immunity in the human population, is generated. This variant is able to cause widespread infection and founds a new cluster of antigenically similar strains.

Influenza viruses can rapidly acquire genetic diversity because of high replication rates in infected hosts, an error-prone RNA polymerase (which introduces mutations during genome replication), and segment reassortment (Figure 2). Mutations that change amino acid residues appear significantly more often than silent mutations in the evolution of the hemagglutinin gene of human influenza A, particularly in the protein epitopes [31]–[34]. This observation indicates that selection for antigenic change of the virus is the driving force in the evolutionary “arms race” between the virus and the immunity of the human population [35]. Reassortment of the eight genome segments between two distinct viruses present simultaneously in a host cell can result in hybrid viruses with genome segments from two different progenitors.

Antigenic mapping allows researchers to generate a quantitative, two-dimensional representation of antigenic distances between genetically divergent strains [26]. This technique has revealed that the relationship between antigenic change and genetic change is nonlinear for the hemagglutinin of influenza A/H3N2. The rate of genetic change of the virus is almost constant over time, but some mutations exert a disproportionately large effect on the antigenic type, whereas others are “hitchhikers” with no phenotypic effect. Elucidating the effects of different mutations at individual sites on the antigenic type will improve our understanding of the overall genotype-to-phenotype mapping for antigenic drift. Furthermore, the antigenic drift of H3N2 is not continuous but punctuated: antigenically homogenous clusters of strains predominate for an average of 3 years before being replaced by a new cluster. In accordance with the punctuated nature of antigenic drift, periods of predominantly neutral evolution alternate with periods of strong selection for antigenic change [13],[36]. Phylogenetic trees illustrating the evolution of the hemagglutinin gene of H3N2 have a cactus-like shape with a strong temporal structure in which the trunk represents the succession of surviving viral lineages over time. Short side branches indicate that most strains are driven to extinction and that viral diversity at any given time is limited [31],[34]. The underlying causes of this punctuated antigenic drift and limited viral diversity at a given point in time have been investigated in phylodynamic modeling studies (Box 2).

**Box 2. Modeling Antigenic Evolution**

There is a long history of the use of mathematical models to study epidemiological and evolutionary systems [63]. For rapidly evolving RNA viruses such as influenza the dynamics of these systems are densely interwoven, and recent work has sought to develop unified “phylodynamic” models to examine the processes underlying the observed epidemiological and evolutionary patterns (reviewed in [35]). A better understanding of the mechanisms driving viral evolution will enhance our capacity to accurately identify novel emerging strains. For influenza, phylodynamic models have been developed to probe the complex processes relating to viral persistence in the human population, antigenic turnover, and the limited genetic diversity at any given point in time. The first models predicted that diversity increases exponentially unless long-term, partial cross-immunity between strains is supplemented by temporary broad immunity that lasts for several months and protects against all infections, regardless of the genetic or antigenic similarity of strains [64],[65]. Subsequently, it has been proposed that a genotype-to-phenotype mapping defined by neutral networks underlies influenza evolution [66]. A neutral network is a set of genotypes linked by single mutations that all map to the same phenotype, in this case the antigenic characteristics of a virus. Hence, genetic divergence is not accompanied by antigenic divergence as long as the genotype remains in the same network. In certain genetic contexts, however, mutations can move a genotype onto an adjacent network, resulting in a significant change in the antigenic phenotype. Incorporating this evolutionary framework into an epidemiological model leads to both epidemiological and evolutionary patterns characteristic of human influenza A/H3N2.

Major changes in antigenicity (antigenic shift) are associated with the introduction of novel viruses into the human population that have a hemagglutinin segment of an influenza A virus from another host species and can be transmitted efficiently among humans [5]. Such viruses may arise by segment reassortment between a human influenza A virus and an influenza A virus from another host species. Alternatively, an entire virus from another host species may cross into the human population. The appearance of such viruses is rare, as it requires the viral genes encoded by the different segments to be compatible with each other and the virus to be capable of replication and transmission in the human population, which is also thought to be a polygenic trait [6],[7],[10],[37],[38]. Antigenic shift can have grave consequences because neutralizing antibodies against the viral surface proteins offer limited or no cross-protection across subtypes. Cross-protection can also be very limited between viruses of the same subtype that have evolved independently in different hosts for long periods of time [14]. Thus, a larger part of the population is susceptible to infection with such viruses than to infection with endemic viruses [10],[14]. Antigenic shift caused three global pandemics in the twentieth century, the 1918 H1N1 pandemic, the 1957 H2N2 pandemic, and 1968 H3N2 pandemic (reviewed in [3]–[5],[8]): The 1918 pandemic had the most devastating impact, with an estimated 20–50 million deaths worldwide [39]. There is some uncertainty concerning the origin of the 1918 virus due to the lack of data from this time [6], [40]–[43]. A recent phylogenetic study suggests that this virus may have been generated by reassortment of avian viruses with already circulating viruses in a mammalian host such as human or swine [44]. The H2N2 virus that caused the 1957 pandemic was a reassortant of five human H1N1 segments and avian segments encoding the viral surface proteins and the PB1 protein. Similarly, the reassortant H3N2 virus of the 1968 pandemic featured avian segments encoding hemagglutinin and PB1. H3N2 still circulates today, together with an H1N1 lineage introduced in 1977, which is similar to the H1N1 viruses circulating in the 1950s [4].

The first pandemic virus of the twenty-first century probably entered the human population in January or February of 2009 [15]. Phylogenetic analyses of the viral genome determined that the virus has a complex reassortment history with segments of “avian-like” Eurasian swine influenza A viruses (NA and M) that were first observed in Eurasian swine in 1979, and of a triple reassortant virus identified in North American swine after 1998. The segments derived from the triple reassortant stem themselves from human H3N2 (PB1), an avian influenza A virus (PA, PB2), and classical North American swine influenza A viruses (HA, NP, NS), which have a common ancestry with the 1918 H1N1 virus [14],[45]. Experiments have shown that the new H1N1 virus replicates efficiently in mammalian model organisms such as ferrets, mice, and cynomolgus macaques and is likely to be capable of long-term circulation in the human population, particularly in the event of further adaptive changes through mutation or reassortment [46]–[48]. The novel H1N1 appears, so far, to cause relatively mild human infections in comparison to other viruses such as the highly pathogenic H5N1 avian influenza A viruses that, since 1997, have repeatedly been transmitted to humans and caused severe disease but so far have not been capable of sustained transmission between humans. The emergence of a novel pandemic virus, which may have been circulating undetected in swine for a decade [14],[45], has highlighted the need for increased genomic surveillance of the viral populations in mammalian hosts such as swine. These hosts could be a vessel for mammalian adaptation of avian viruses, either by reassortment with human or swine viruses or through adaptive changes [8], but have been monitored less intensely than avian populations. The latest emergence of a pandemic H1N1 virus has also underscored the vital importance of further research into the molecular factors that determine the host range and capacity for sustained human-to-human transmission of influenza A viruses.

## Reassortment in Subtype Evolution

Whole-genome studies have revealed that segment reassortment between different viruses of the same subtype is an important mechanism in the evolution of human-adapted subtypes and generates extensive genome-wide diversity [34], [36], [49]–[51]. Periodic selective sweeps caused by a novel antigenic drift variant rising to predominance reduce the genomic diversity of the circulating viral population, either genome-wide or for the hemagglutinin segment only [12]. Reassortment results in substantial differences in the evolutionary histories of individual segments. However, similarities in the histories of some segments indicate that besides the antigenic characteristics of hemagglutinin, the genomic context and compatibility of certain segment combinations might be an important contributor to viral fitness [12],[51]. A case in point is the antigenically novel “Fujian” strain which became predominant in the 2003–2004 season, following a reassortment event that placed a hemagglutinin segment from a lineage that had been circulating at low levels for several years into a new genomic context [49]. The importance of other segments in the adaptive evolution of the virus is further supported by the observation that a number of other segments, including the one encoding neuraminidase, evolve at similar rates to the segment encoding hemagglutinin [12].

## Geographic Spread

Genomic analysis has led to profound insights into the global patterns of circulation and evolution of influenza A. Over the course of seasonal epidemics in temperate regions, little evidence has been found for selection for amino acid change and adaptive evolution in the antigenic regions of the surface proteins [36]. There is, however, substantial genetic diversity due to multiple introductions of distinct strains, wide spatial spread, and frequent segment reassortment in seasonal epidemics [9],[12],[36],[49],[50]. The viral population circulating in one season does not directly seed the epidemic in the following one. Instead, gene flow and viral spread are global, with similar strains appearing in northern and southern hemisphere epidemics across several seasons. There is a global reservoir of viral diversity from which seasonal epidemics in temperate regions are seeded [12],[27],[52]. This reservoir is located in East-Southeast Asia, where a region-wide network of temporally overlapping epidemics maintains infection incidence throughout the year [27]. Novel strains appear in this region on average 6–9 months before they emerge in Oceania, Europe, and North America and 12–18 months before they reach South America.

## Challenges for the Future

A key objective for research into the antigenic drift of influenza A is to improve the accuracy of vaccine strain choice, in particular for seasons preceding the establishment of novel antigenic drift variants. More intensive surveillance and sampling, particularly in East-Southeast Asia, could facilitate the early detection of novel emerging drift variants and alleviate problems related to the time required for vaccine production. A better understanding of the evolutionary and epidemiological rules governing antigenic drift, viral fitness, the role of the source region, and establishment of predominance would be particularly helpful for the selection of vaccine strains when considerable variation among antigenically novel strains is observed and it is unclear which, if any, will become predominant. Such insights are likely to come both from phylodynamic modeling studies and by mining genomic resources for genome-wide properties associated with viral fitness and predominance. Some molecular properties of hemagglutinin with predictive value for this task have already been identified [53]–[56], such as the number of changes at sites under positive selection or in the most extensively altered epitope, although the sites under selection might change over time [26]. It is notable that the lack of antigenic information for sequenced viral isolates in public repositories currently restricts the direct analysis of genetic determinants in antigenic drift [24]. If the World Health Organization were to establish similar policies for the deposition of antigenic information into public databases as exist for sequence data, this could create a valuable resource for research in this area. As existing databases grow, new statistical and computational techniques are being developed for interpretation of these large-scale, population-level genomic datasets in combination with epidemiological and phenotypic information [57]. Ultimately, the expert analysis of the WHO in the detection and control of antigenically novel emerging strains could be extensively supported by the development of a suitable predictive framework based on statistical learning that takes into consideration the population-level phylodynamics of antigenic change [57],[58]. Such a framework could utilize epidemiological, genomic, and antigenic information and detailed knowledge of the genetic and epidemiological characteristics of antigenic drift to assess the likelihood of strains rising to predominance.
